# Antivirulence Strategies for the Treatment of *Staphylococcus aureus* Infections: A Mini Review

**DOI:** 10.3389/fmicb.2020.632706

**Published:** 2021-01-14

**Authors:** Caleb A. Ford, Ian M. Hurford, James E. Cassat

**Affiliations:** ^1^Department of Biomedical Engineering, Vanderbilt University, Nashville, TN, United States; ^2^Department of Pediatrics, Division of Pediatric Infectious Diseases, Vanderbilt University Medical Center, Nashville, TN, United States; ^3^Department of Pathology, Microbiology, and Immunology, Vanderbilt University Medical Center, Nashville, TN, United States; ^4^Vanderbilt Center for Bone Biology, Vanderbilt University Medical Center, Nashville, TN, United States; ^5^Vanderbilt Institute for Infection, Immunology, and Inflammation (VI4), Vanderbilt University Medical Center, Nashville, TN, United States

**Keywords:** *Staphylococcus aureus* – bacteria, antivirulence, antimicrobial resistance, virulence, infection, quorum sensing, accessory gene regulator, toxin

## Abstract

*Staphylococcus aureus* is a Gram-positive bacterium capable of infecting nearly all host tissues, causing severe morbidity and mortality. Widespread antimicrobial resistance has emerged among *S. aureus* clinical isolates, which are now the most frequent causes of nosocomial infection among drug-resistant pathogens. *S. aureus* produces an array of virulence factors that enhance *in vivo* fitness by liberating nutrients from the host or evading host immune responses. Staphylococcal virulence factors have been identified as viable therapeutic targets for treatment, as they contribute to disease pathogenesis, tissue injury, and treatment failure. Antivirulence strategies, or treatments targeting virulence without direct toxicity to the inciting pathogen, show promise as an adjunctive therapy to traditional antimicrobials. This Mini Review examines recent research on *S. aureus* antivirulence strategies, with an emphasis on translational studies. While many different virulence factors have been investigated as therapeutic targets, this review focuses on strategies targeting three virulence categories: pore-forming toxins, immune evasion mechanisms, and the *S. aureus* quorum sensing system. These major areas of *S. aureus* antivirulence research demonstrate broad principles that may apply to other human pathogens. Finally, challenges of antivirulence research are outlined including the potential for resistance, the need to investigate multiple infection models, and the importance of studying antivirulence in conjunction with traditional antimicrobial treatments.

## Introduction

*Staphylococcus aureus*, a Gram-positive bacterium, asymptomatically colonizes approximately 30% of the population and can infect nearly every tissue in the body (Wertheim et al., 2005; Monaco et al., 2017). *S. aureus* readily adapts its metabolic and virulence responses in different tissues, causing superficial (e.g., folliculitis) and invasive infections (e.g., osteomyelitis; Balasubramanian et al., 2017; Potter et al., 2020). *S. aureus* biofilm formation, toxin production, and immune evasion strategies limit host antibacterial immune responses (Thammavongsa et al., 2015a; Muthukrishnan et al., 2019). Therefore, staphylococcal infections often necessitate long-term antibiotics (Lew and Waldvogel, 2004; Hatzenbuehler and Pulling, 2011). However, widespread antimicrobial resistance has highlighted the need to develop additional treatments (Ventola, 2015).

*Staphylococcus aureus* is the leading cause of nosocomial infections among antibiotic-resistant organisms, making staphylococcal infections a major target for investigation of antivirulence therapies (Sievert et al., 2013; Dickey et al., 2017). For the purposes of this review, antivirulence therapies are defined as those that do not inhibit bacterial growth *in vitro* but limit the production or function of virulence factors that promote infection or incite host damage *in vivo*. Antivirulence strategies aim to mitigate host tissue damage as host immune responses or conventional antimicrobials eradicate infection.

**FIGURE 1:**
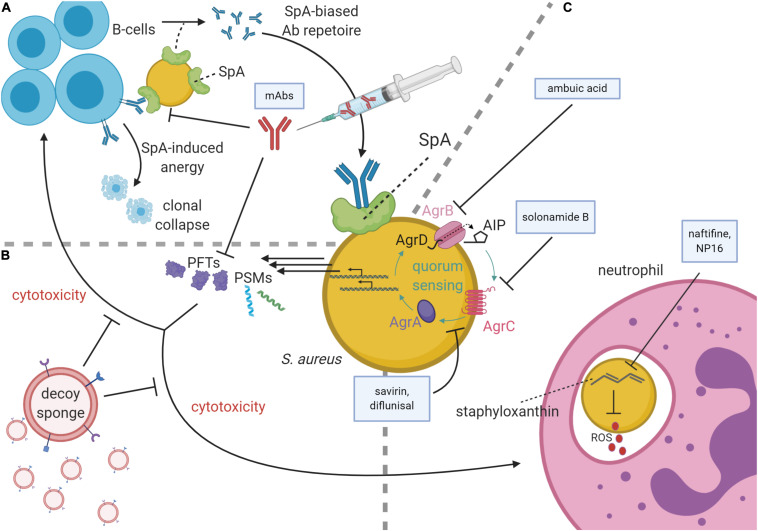
*Staphylococcus aureus* virulence pathways and antivirulence strategies. **(A)** At top left, B-cells secrete antibodies against *S. aureus* antigens, the Fc region of which may be bound by Staphylococcal protein A (SpA) on the *S. aureus* membrane, thereby subverting immune responses. SpA may also bind the Fab portion of V_H_3 family B-cell receptors, causing SpA-induced clonal expansion and anergic collapse. Superantigen activity of SpA also influences antibody (Ab) production by narrowing the breadth of anti-staphylococcal antibodies from V_H_3 family B-cells, creating a preference for poorly functioning anti-SpA clones. To inhibit SpA activity, therapeutic monoclonal antibodies (mAbs) raised against an attenuated SpA have been delivered parenterally. **(B)** mAbs can also inhibit pore-forming toxins (PFTs). PFTs and phenol-soluble modulins (PSMs) are cytolytic toxins regulated by the accessory gene regulator (*agr*) quorum sensing system. To diminish these toxins’ cytotoxicity, decoy sponges limit the activity of the PFTs by presenting a variety of decoy receptors on their surfaces. **(C)** Neutrophils use reactive oxygen species (ROS) to kill phagocytosed *S. aureus*. These ROS are inhibited by staphyloxanthin, which in turn can be inhibited by the drugs naftifine and NP16. At the image center, a schematic of the *agr* quorum sensing system is shown with color-coded protein labels. Beginning with transcription and translation of the *agr* operon, AgrB modifies and secretes AgrD to produce autoinducing peptide (AIP). Upon reaching quorum, AIP binds the receptor kinase, AgrC, which phosphorylates the response regulator, AgrA. AgrA activates the P2 and P3 promoters of the *agr* operon in a positive feedback loop and increases the production of many cytolytic virulence factors including PSMs and many PFTs. The Agr system may be targeted by several agents including ambuic acid (inhibition of AIP secretion), solonamide B (inhibition of AIP activation of AgrC), and savirin and diflunisal (inhibition of AgrC and AgrA downstream of AIP sensing).

The number of published studies on antivirulence techniques has increased dramatically over the last decade (Maura et al., 2016; Dickey et al., 2017). While most antivirulence strategies for staphylococcal disease are currently preclinical, several antibody-based antivirulence approaches and one immunomodulatory peptide (NCT02469857, ClinicalTrials.gov [Internet], 2015) are in clinical trials (Huynh et al., 2016; Levy et al., 2016; François et al., 2018, 2019; Magyarics et al., 2019). This review will focus on recent investigations into antivirulence strategies targeting key virulence mechanisms of *S. aureus* as outlined in Figure 1 and listed in Table 1.

**TABLE 1:** Each of the virulence mechanisms of *S. aureus* discussed within the Mini Review is listed with accompanying drug candidates as shown.

Virulence factors	Treatment strategies	Drug candidates
**Pore-forming toxins**		
α-hemolysin (Hla)	mAbs, decoy sponges	MEDI4893^1^, AR-301^2^, ASN100*^3^
Panton-Valentine leukocidin	mAbs, decoy sponges	ASN100*^3^
Leukocidin AB (LukAB, LukGH)	mAbs, decoy sponges	ASN100*^3^
**Immune cell evasion**		
Staphyloxanthin	Small molecule inhibitors	Naftifine^4^, NP16^5^
Staphylococcal protein A (SpA)	mAbs, vaccines	Anti-SpA_*KKAA*_ mAbs^6^, rFSAV^†7^
**Accessory gene regulator: quorum sensing**		
AIP production	Small molecule inhibitors	Ambuic acid^8^
AIP sensing by AgrC	Small molecule inhibitors	Solonamide B^9^
AgrC→AgrA signaling	Small molecule inhibitors	Diflunisal^10^, Savirin^11^

*^1^François et al., 2019;*

*^2^François et al., 2018;*

*^3^Magyarics et al., 2019;*

*^4^Chen et al., 2016;*

*^5^Gao et al., 2017;*

*^6^Kim et al., 2012;*

*^7^Zeng et al., 2020a;*

*^8^Nakayama et al., 2009;*

*^9^Mansson et al., 2011;*

*^10^Khodaverdian et al., 2013;*

^11^Sully et al., 2014;

*two mAbs, and ^†^five antigens.

## Antivirulence Strategies

### Antivirulence Targeting of Pore-Forming Toxins

*Staphylococcus aureus* produces an arsenal of pore-forming toxins (PFTs) that kill host cells, thereby combatting immune responses and liberating nutrients from the host. For more information regarding PFTs and their pharmacologic targeting, readers are directed to PFT-specific reviews (Reyes-Robles and Torres, 2017; Escajadillo and Nizet, 2018). Here, we highlight antivirulence approaches to three major PFTs: α-hemolysin (Hla, α-toxin), Panton-Valentine leukocidin (PVL), and leukocidin AB (LukAB, also known as LukGH). Hla was the first recognized PFT and is regarded as a key virulence factor of *S. aureus* (Bhakdi and Tranum-Jensen, 1991). PVL is highly cytotoxic, particularly when present during invasive infections such as necrotizing pneumonia (Panton and Valentine, 1932; Shallcross et al., 2013; Hu et al., 2015). LukAB is a recently identified bi-component leukocidin and one of the most immunogenic toxins in terms of neutralizing antibody induction (Thomsen et al., 2014). Expression of all three aforementioned PFTs is regulated by RNAIII, a major effector of the accessory gene regulator (*agr*) quorum sensing operon of *S. aureus*. Therefore, many strategies employed to inhibit these toxins target the *agr* system, which is discussed later (Le and Otto, 2015). Two primary approaches target PFTs directly: neutralizing antibodies and decoy receptors.

MEDI4893 (suvratoxumab), an Hla-neutralizing monoclonal antibody (mAb) previously known as LC10, is one of the most well-characterized antivirulence therapies for treating *S. aureus* infection. Hla binds the metalloprotease ADAM10 to promote oligomerization and pore formation (Wilke and Wardenburg, 2010). Exploiting this mechanism, MEDI4893 inhibits Hla interactions with ADAM10 and Hla self-oligomerization by binding a highly conserved region of Hla (Wilke and Wardenburg, 2010; Foletti et al., 2013; Oganesyan et al., 2014). Alanine scanning of this highly conserved region identified *S. aureus* mutants resistant to MEDI4893 neutralization. Importantly, mutants resistant to neutralization show reduced dermatonecrosis and mortality in murine models of *S. aureus* skin infection and pneumonia, respectively, likely reflecting impaired Hla function in the mutants (Tkaczyk et al., 2018). As of November 2020, MEDI4893 has completed Phase 2 clinical trials for prevention of *S. aureus* pneumonia in high-risk ICU patients. The data demonstrate a non-statistically significant trend toward prevention [relative risk reduction of 31.9% (90% CI, -7.5% to 56.8%)] and a favorable safety profile (Yu et al., 2017; François et al., 2019). Several other neutralizing antibodies against Hla have reached clinical trials, including AR-301 and ASN100. AR-301 is a mAb targeting Hla with previously published safety data that has now entered Phase 3 trials as an adjuvant therapy for *S. aureus* pneumonia (NCT03816956; François et al., 2018; ClinicalTrials.gov [Internet], 2019). ASN100 is a cocktail of two mAbs that together neutralize six cytolytic toxins of *S. aureus* including Hla, PVL, and LukAB, along with three other bicomponent toxins: γ-hemolysin AB (HlgAB), γ-hemolysin CB (HlgCB), and leukocidin ED (LukED). ASN100 limits tissue damage in a rabbit model of *S. aureus* pneumonia and completed Phase 1 clinical safety trials (Magyarics et al., 2019; Stulik et al., 2019). Notably, however, a Phase 2 trial for ASN100 was terminated due to futility (NCT02940626, ClinicalTrials.gov [Internet], 2016). mAbs targeting LukAB have been shown to improve bacterial burdens in murine models of systemic infection (Thomsen et al., 2017). Furthermore, inactivation of LukAB improves adaptive immune responses, as LukAB-mediated lysis of CD11b-positive antigen presenting cells attenuates adaptive responses (DuMont et al., 2013; Berends et al., 2019). LukAB does not readily target murine cells since LukAB, like many *S. aureus* toxins, demonstrates strong tropism for human cells, limiting its investigation in animal models (Spaan et al., 2017). Related bicomponent toxins, LukED and HlgAB, target similar cells in mice as LukAB does in humans, making LukED and HlgAB more impactful in preclinical mouse models (Spaan et al., 2017). In mice, immunization with LukED and HlgAB antigens induces stronger anti-LukED and anti-HlgAB antibody responses relative to infection with wild-type *S. aureus* and improves survival upon subsequent challenge with *S. aureus* (Tam et al., 2020).

In addition to neutralizing antibodies, decoy receptors have been proposed as treatments for staphylococcal infection. Hla can self-oligomerize into an amphiphilic, pore-forming state in the presence of deoxycholate micelles (a bile salt related to cholesterol; Bhakdi et al., 1982). Sphingomyelin-cholesterol micelles were previously shown to act as decoy sponges that inhibit Hla toxicity *in vitro* and in a murine sepsis model (Henry et al., 2015). Recently, exosomes (termed “defensosomes”) with increased ADAM10 were found to be secreted from host cells in a TLR-dependent manner in response to *S. aureus*, resulting in Hla sequestration and a reduction in disease mortality (Keller et al., 2020). As a therapeutic, poly(lactic-co-glycolic acid) (PLGA)-based nanoparticles coated with natural membranes of human RBCs fulfill a similar decoy mechanism to combat infection (Chen et al., 2018). These “toxin nanosponges” are coated with human RBC membranes agnostic of intended receptors, and inhibit the activity of Hla and other toxins. In summary, therapeutics developed for direct inhibition of PFTs have thus far exploited two naturally occurring mechanisms: neutralizing antibodies and decoy membrane receptors.

### Antivirulence Strategies Targeting Immune Evasion Mechanisms

#### Staphyloxanthin

Staphyloxanthin is an antioxidant that gives *S. aureus* its eponymous golden color and protects against reactive oxygen species (ROS) produced by innate immune cells *in vivo* (Marshall and Wilmoth, 1981; El-Agamey et al., 2004; Liu et al., 2005; Clauditz et al., 2006). The genes responsible for staphyloxanthin synthesis are in the *crtOPQMN* operon, which is highly conserved across *S. aureus* strains, making it an attractive antivirulence target (Pelz et al., 2005). Two commonly targeted enzymes are CrtM and CrtN (Wieland et al., 1994). CrtM is analogous to a squalene synthase in human cholesterol biosynthesis, and many compounds inhibit both enzymes (Liu et al., 2008; Song et al., 2009). Inhibition of *S. aureus* CrtM with repurposed lipid-lowering medications targeting squalene synthase may be favorable in infected patients with hyperlipidemia (Sharma et al., 1998; Liu et al., 2008; Song et al., 2009; Lin et al., 2010). However, due to the potential detrimental effects of human squalene synthase inhibition when targeting CrtM, CrtN may be a more attractive target for inhibiting staphyloxanthin biosynthesis (Gao et al., 2017). Naftifine, an FDA-approved antifungal, and its related analogs were the first identified inhibitors of CrtN (Mühlbacher, 1991). Naftifine inhibition of staphyloxanthin production sensitizes *S. aureus* to ROS and limits mortality in a murine sepsis model (Chen et al., 2016). Recent studies have also identified NP16 and naftifine derivatives that inhibit CrtN with improved potency and reduce *S. aureus* bacterial burdens during systemic infection in mice (Gao et al., 2017; Li et al., 2018).

#### Staphylococcal Protein A

Staphylococcal protein A (SpA) is a cell wall-anchored protein which contains immunoglobulin-binding domains that bind the Fcγ portion of human IgG antibodies and the Fab of some IgM subtypes (Kronvall and Williams, 1969; Sjödahl, 1977; Moks et al., 1986; Roben et al., 1995; Graille et al., 2000). SpA binding of the Fcγ portion of IgG limits antibody-mediated phagocytosis (Forsgren and Nordström, 1974; Forsgren and Quie, 1974; Thammavongsa et al., 2015a). SpA also has superantigen activity by crosslinking of V_H_3 B-cell receptors, thereby inducing B-cell clones of limited breadth that produce poorly effective anti-staphylococcal antibodies as well as anergic B-cell populations (Roben et al., 1995; Pauli et al., 2014; Thammavongsa et al., 2015a; Sun et al., 2018). SpA is almost universally present in clinical *S. aureus* strains and likely contributes to vaccine failure and attenuation of mAb-based treatments (Forsgren, 1970; Kim et al., 2010; Bagnoli et al., 2012).

Despite the subversion of antibody-mediated immune responses, antivirulence strategies targeting SpA have primarily focused on antibody-based treatments. By developing a mutant of SpA unable to bind immunoglobulins (SpA_KKAA_), Kim and colleagues demonstrated that, following exposure to the SpA_KKAA_ mutant, mice develop opsonizing antibodies and reduced bacterial burdens upon rechallenge with wild-type *S. aureus* (Kim et al., 2010; Falugi et al., 2013). Passive immunization with anti-SpA_KKAA_ mAbs neutralizes SpA activity in systemic *S. aureus* infection models and maintains efficacy when humanized by grafting the complementary determining region onto human IgG1 (Kim et al., 2012; Thammavongsa et al., 2015b). Additionally, passive neutralization of SpA encourages production of effective anti-staphylococcal antibodies that promote bacterial decolonization from nasal and gastrointestinal mucosa of mice (Chen et al., 2019). Furthermore, humanized anti-SpA_KKAA_ antibodies promote bacterial clearance upon binding SpA through the induction of the complement cascade, and effective complement activation is dependent upon the glycosylation of SpA-binding mAbs (Chen et al., 2020). SpA_KKAA_ has been proposed as part of a four-antigen *S. aureus* vaccine that demonstrates improved survival in systemic *S. aureus* infection in mice (Kim et al., 2011; Rauch et al., 2014). Moreover, a five-antigen vaccine (rFSAV), which includes SpA_KKAA_, has completed a Phase 1 trial and induces high antibody titers (Zeng et al., 2020a,b).

### Antivirulence Strategies Targeting Quorum Sensing

The primary regulatory pathway studied as an antivirulence target in *S. aureus* has been the *agr* system. The proteins of the *agr* operon enable *S. aureus* quorum sensing and regulate many virulence factors including PFTs and phenol-soluble modulins (PSMs; Le and Otto, 2015). PSMs are a class of cytotoxic alpha-helical peptides and are one of the few toxins to be directly regulated by the system’s response regulator, AgrA (Queck et al., 2008; Cheung et al., 2014). Given its broad impact on virulence factor production, targeting the *agr* system as an antivirulence treatment has been reviewed extensively (reviewed in Khan et al., 2015, and Piewngam et al., 2020). The *agr* quorum sensing system requires production and sensing of autoinducing peptide (AIP). Factors that inhibit *agr* can inhibit any step in AIP production, sensing, and subsequent transcriptional activation of the *agr* P2 and P3 promoters, as well as the promoter for PSMs (Thoendel et al., 2011; Khan et al., 2015). For example, ambuic acid and solonamide B inhibit AIP production and sensing, respectively, while savirin inhibits downstream transcriptional activity of AgrA (Nakayama et al., 2009; Mansson et al., 2011; Sully et al., 2014). *S. aureus* contains four distinct AIP peptide sequences that exist in 3 cross-inhibition groups: I/IV, II, and III (Ji et al., 1997; Jarraud et al., 2000). I and IV differ by one amino acid and cross-activate (Jarraud et al., 2000). Otherwise, the presence of alternative AIP sequences from any other *agr* group competitively inhibits AIP autorecognition (Ji et al., 1997). This phenomenon has been observed for AIP peptides from many other staphylococcal species, which produce AIP molecules that cross-inhibit *S. aureus agr* signaling (Otto et al., 2001; Canovas et al., 2016, Canovas et al., 2017; Williams et al., 2019).

Treatment with AIP analogs has been investigated as an antivirulence mechanism using rational design and high-throughput screening mechanisms (reviewed in Horswill and Gordon, 2020). Changing native amino acid residues or the thiolactone ring of AIP can increase or decrease *agr* signaling (Mayville et al., 1999; Otto et al., 2001; Lyon et al., 2002). Substitution of the AIP-III thiolactone ring with a lactam was demonstrated to inhibit AIP binding for all four *agr* types of *S. aureus* in the nanomolar range *in vitro* but has not yet been tested *in vivo* (Tal-Gan et al., 2016). High-throughput screening also elucidated natural compounds that competitively inhibit AIP binding to its receptor kinase, AgrC. One of the first discovered natural inhibitors of *agr* signaling was solonamide B, a cyclodepsipeptide from *Photobacterium halotolerans* (Mansson et al., 2011). Solonamide B and its derivatives inhibit binding of AIP to AgrC, and through this mechanism, solonamide B mitigates toxin-mediated cytolysis of phagocytes and δ-toxin-mediated atopic dermatitis in mice (Nielsen et al., 2014; Baldry et al., 2016, 2018). Since the discovery of solonamide B, other compounds that inhibit AIP binding to AgrC have been discovered. For example, *agr*-inhibiting lipoproteins from *Bacillus subtilis* promote decolonization in mice, possibly through *agr*-regulated adhesins. Furthermore, colonization with probiotic *Bacillus* species is inversely related to *S. aureus* colonization in the gastrointestinal tract and nares in humans, corroborating the findings in mice (Piewngam et al., 2018).

Inhibitors of *agr* also target signaling downstream of the receptor kinase (AgrC) and response regulator (AgrA). Savirin is a well-characterized antivirulence compound that targets AgrA by interfering with its transcriptional regulation (Sully et al., 2014). In an air-pouch infection model, mice treated with savirin exhibit reduced dermatonecrosis. While most tested *agr* inhibitors are administered at the time of bacterial inoculation, savirin was tested in a delayed treatment model and still showed efficacy, though the effect size was reduced compared to immediate treatment (Sully et al., 2014). An important future direction is therefore to evaluate the efficacy of antivirulence compounds in delayed treatment models, which perhaps more accurately reflect the clinical scenario in humans. In addition to novel small molecules, existing drugs have been identified that target AgrA and are amenable to drug repurposing. For example, diflunisal is an FDA-approved non-steroidal anti-inflammatory drug (NSAID) predicted to inhibit phosphorylation of AgrA by AgrC (Hannah et al., 1977; Khodaverdian et al., 2013). Diflunisal is a derivative of salicylic acid, which also has antivirulence properties through effects on Sigma Factor B (SigB) activity (Kupferwasser et al., 2003). In a murine model of osteomyelitis, delivery of diflunisal mitigates infection-mediated bone loss without impacting *S. aureus* burden in the infected femur (Hendrix et al., 2016; Ford et al., 2020; Spoonmore et al., 2020).

Targeting of *agr* quorum sensing is controversial in part because of its role in regulating biofilm dispersal (Boles and Horswill, 2008). Inactivation of *agr* in *S. aureus* promotes biofilm formation *in vitro* and impairs biofilm dispersal during osteomyelitis (Vuong et al., 2000; Nishitani et al., 2015). Biofilms are often associated with chronic infections, and *agr*-deficient mutants have emerged during the course of biofilm-associated infections (Brady et al., 2008; Traber et al., 2008; Suligoy et al., 2018; He et al., 2019). Therefore, it is important to test antivirulence compounds targeting *agr* signaling in the context of non-biofilm and biofilm infections as *agr* inhibition may lessen toxin-mediated virulence at the risk of promoting infection persistence (He et al., 2019). Additionally, it is important to recognize the natural occurrence of *agr*-deficient isolates, for which *agr* inhibition is inherently ineffective.

## Conclusion

Antivirulence strategies are actively under preclinical investigation as therapies for staphylococcal disease. Several mAbs have advanced to clinical trials (e.g., AR-301 in phase 3, NCT03816956, ClinicalTrials.gov [Internet], 2019) and may soon be used as adjunctive therapies with conventional antimicrobials. We are unaware of any clinical trials in the United States that have investigated the use of small molecule inhibitors as antivirulence agents for *S. aureus* infection as of November 2020. However, several investigational antivirulence compounds are FDA-approved for alternative purposes. For example, diflunisal, an NSAID, inhibits the *agr* system, and terbinafine, an antifungal, inhibits staphyloxanthin production (Khodaverdian et al., 2013; Chen et al., 2016). Drug repurposing may therefore offer an attractive timeline for translation compared to the timeline for developing novel compounds.

Some traditional antibiotics also exhibit antivirulence properties. At sub-inhibitory concentrations, azithromycin inhibits toxin-mediated hemolysis *in vitro* and limits tissue destruction in a model of *S. aureus* keratitis (Ikemoto et al., 2020). Additionally, the bacteriostatic antimicrobial clindamycin is a protein synthesis inhibitor that suppresses toxin production *in vitro* and is now recommended for treatment of toxin-mediated diseases (e.g., toxic shock syndrome; Schlievert and Kelly, 1984; Ohlsen et al., 1998; Stevens et al., 2007; Nathwani et al., 2008). However, different antibiotic classes alter virulence factor production in distinct ways. Treatment of *S. aureus* with β-lactam antibiotics is thought to increase virulence factor production, including that of Hla and PVL (Ohlsen et al., 1998; Stevens et al., 2007). Recognizing that treatment with some antibiotics may increase virulence underscores the importance of investigating antivirulence strategies and how these will partner with traditional treatment regimens.

Unlike traditional antimicrobials, antivirulence therapies, strictly defined, do not inhibit bacterial growth *in vitro* and likely have decreased risk of resistance development. However, *in vitro* environments do not recapitulate elements of the host immune response found *in vivo*. Since virulence factors promote bacterial survival in the host, some selective pressure for resistance likely exists (Maura et al., 2016). Emergent resistance to antivirulence therapeutics has been observed (Maeda et al., 2012). However, not all antivirulence strategies carry the same risk of resistance. Targeting individual virulence mechanisms, such as PFTs, may have more limited risk of resistance compared to regulatory targets like *agr* that have the potential to impact large sets of virulence factors. Moreover, *S. aureus* mutants with emergent resistance to some antivirulence treatments may exhibit reduced overall fitness, as has been observed with mutants resistant to MEDI4893 (Tkaczyk et al., 2018). Targeting individual PFTs in *S. aureus* may lack efficacy due to functional redundancy of cytolytic toxins. Unfortunately, the influence of such redundancy in preclinical investigations may be missed due to the species tropism of many *S. aureus* toxins. For example, unlike LukED and HlgAB, PVL has no cytotoxic effect on murine cells but impacts invasive infection clinically (Spaan et al., 2017). Animal models continue to elucidate fundamental host-pathogen interactions; however, many otherwise promising *S. aureus* therapies demonstrate strong preclinical data without success in clinical trials (Bagnoli et al., 2012). Because of the high costs and risks of clinical trials, improved animal models offer an attractive compromise. Humanized mice with engrafted human immune cells may advance the translatability of mouse models of *S. aureus* infection (Allen et al., 2019). Virulence of human-targeted toxins (such as PVL) is more likely to be revealed in mice with human hematopoietic cells (Mrochen et al., 2020). Improved animal models may help determine which therapeutics should advance to clinical trials as antivirulence therapies continue to be explored.

## Author Contributions

CF, IH, and JC contributed to initial conceptualization of the review. CF and IH performed initial literature reviews and manuscript drafting. JC contributed to literature review and extensive manuscript editing. All authors contributed to the article and approved the submitted version.

## Conflict of Interest

The authors declare that the research was conducted in the absence of any commercial or financial relationships that could be construed as a potential conflict of interest.
